# Supplementary material for: Deepening the Mechanisms of Visceral Pain Persistence: An Evaluation of the Gut-Spinal Cord Relationship
Source: Cells. 2020 Jul 24;9(8):1772. doi: 10.3390/cells9081772 (PMC7464824; doi:10.3390/cells9081772)
Supplement: Supplementary file 1 [file cells-09-01772-s001.pdf]

cells

MDPI

Supplementary Table S1. Antibodies and reactives used for immunoenzymatic and immunofluorescence histochemistry.

Primary antibodies (antigen)	Species	Dilution	Incubation time	Source
SP	rabbit	1:15000 (immunoperox) 1:5000 (immunoflu)	o.n	Millipore, Temecula, CA, USA
MHC-II	mouse	1:20	o.n.	Abcam, Cambridge, UK
Iba1	rabbit	1:250	o.n.	Wako, Richmond, VA, USA
GFAP	rabbit	1:500	o.n.	Dako,Carpinteria, CA, USA
**Secondary antibodies and reagents**				
Biotinylated anti-rabbit IgG	goat	1:200	1 hour	Vector Lab, Burlingame, USA
VectastainABC Elite kit			30 min	Vector Lab, Burlingame, USA
Alexa Fluor R 488 F(ab') 2 fragment of goat anti rabbit	goat	1:300	1 hour	Molecular Probes, Eugene, Oregon, USA
Biotinylated anti-mouse IgG (H+L) affinity purified	goat	1:300	1 hour	Vector Laboratories, Inc, Burlingame, CA
Streptavidin Alexa Fluor 555conjugated		1:300	1 hour	Molecular Probes, Eugene, USA
TO-PRO3			5 min	Invitrogen, Eugene, USA
Alexa Fluor® Plus 488 goat anti rabbit IgG	goat	1:500	1 hour	Invitrogen, Eugene, USA
Alexa Fluor® Plus 568 goat anti rabbit IgG	goat	1:500	1 hour	Invitrogen, Eugene, USA
DAPI (4', 6-diamidin-2-fenilindolo, dihydrochloride)		1:2000	1 hour	Molecular Probes, Eugene, USA

Supplementary Table S2. Total, resting and activated cells count concerning immunofluorescence staining results expressed in Figure 7.

		GFAP-positive cells		***Iba1-positive cells***	
		**Dorsal horn**	**Ventral horn**	**Dorsal horn**	**Ventral horn**
Ctrl (n = 40)	**Total cells count** (mean ± S.E.M.)	60.42 ± 8.19	119.96 ± 7.77	21.42 ± 1.27	24.96 ± 0.88
	**Resting cells count** (mean ± S.E.M.)	55.21 ± 8.05	109.96 ± 7.81	17.17 ± 1.37	18.88 ± 1.02
	**Activated cells count** (mean ± S.E.M.)	5.21 ± 0.37	10 ± 0.88	4.25 ± 0.39	6.08 ± 0.45
DNBS (n = 40)	**Total cells count** (mean ± S.E.M.)	78.78 ± 5.36	138.09 ± 6.65	17.52 ± 0.66	25.17 ± 0.68
	**Resting cells count** (mean ± S.E.M.)	57.85 ± 5.58	119.82 ± 5.87	5.77 ± 0.61	15.32 ± 0.74
	**Activated cells count** (mean ± S.E.M.)	20.93 ± 1.23	18.27 ± 1.86	11.75 ± 0.71	9.85 ± 0.63
